# (*E*)-*N*′-[1-(Thio­phen-2-yl)ethyl­idene]benzohydrazide

**DOI:** 10.1107/S1600536811033101

**Published:** 2011-08-27

**Authors:** Shang Shan, Yan-Lan Huang, Han-Qi Guo, Deng-Feng Li, Jian Sun

**Affiliations:** a College of Chemical Engineering and Materials Science, Zhejiang University of Technology, People’s Republic of China

## Abstract

The title compound, C_13_H_12_N_2_OS, was obtained from the condensation reaction of 2-acetyl­thio­phene and benzohydrazide. In the mol­ecule, the formohydrazide fragment is approximately planar (r.m.s deviation = 0.0146 Å) and the mean plane is oriented at dihedral angles of 24.47 (11) and 28.86 (13)°, respectively, to the phenyl and thio­phene rings. The thio­phene and phenyl rings make a dihedral angle of 53.21 (8)°. The benzamide fragment and thio­phene ring are located on the opposite sides of the C=N bond, showing an *E* conformation. Classical inter­molecular N—H⋯O hydrogen bonds and weak C—H⋯O inter­actions are present in the crystal structure: three such bonds occur to the same O-atom acceptor.

## Related literature

For applications of hydrazone derivatives in the biological field, see: Okabe *et al.* (1993 ▶). For general background to this work, see: Qiang *et al.* (2007 ▶). For a related structures, see: Xia *et al.* (2009 ▶); Shan *et al.* (2011 ▶)
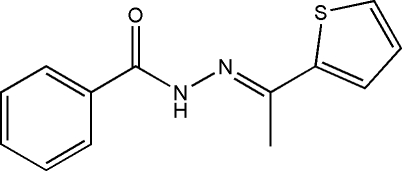


## Experimental

### 

#### Crystal data

C_13_H_12_N_2_OS
*M* *_r_* = 244.31
Orthorhombic, 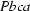

*a* = 9.906 (3) Å
*b* = 10.542 (5) Å
*c* = 22.870 (5) Å
*V* = 2388.3 (14) Å^3^
*Z* = 8
Mo *K*α radiation
μ = 0.26 mm^−1^
*T* = 294 K
0.32 × 0.29 × 0.28 mm

#### Data collection

Rigaku R-AXIS RAPID IP diffractometer
7859 measured reflections
2153 independent reflections
1552 reflections with *I* > 2σ(*I*)
*R*_int_ = 0.036

#### Refinement

*R*[*F* ^2^ > 2σ(*F* ^2^)] = 0.041
*wR*(*F* ^2^) = 0.101
*S* = 1.03
2153 reflections
155 parameters
H-atom parameters constrained
Δρ_max_ = 0.20 e Å^−3^
Δρ_min_ = −0.17 e Å^−3^

### 

Data collection: *PROCESS-AUTO* (Rigaku, 1998 ▶); cell refinement: *PROCESS-AUTO*; data reduction: *CrystalStructure* (Rigaku/MSC, 2002 ▶); program(s) used to solve structure: *SIR92* (Altomare *et al.*, 1993 ▶); program(s) used to refine structure: *SHELXL97* (Sheldrick, 2008 ▶); molecular graphics: *ORTEP-3 for Windows* (Farrugia, 1997 ▶); software used to prepare material for publication: *WinGX* (Farrugia, 1999 ▶).

## Supplementary Material

Crystal structure: contains datablock(s) I, global. DOI: 10.1107/S1600536811033101/xu6116sup1.cif

Structure factors: contains datablock(s) I. DOI: 10.1107/S1600536811033101/xu6116Isup2.hkl

Supplementary material file. DOI: 10.1107/S1600536811033101/xu6116Isup3.cml

Additional supplementary materials:  crystallographic information; 3D view; checkCIF report

## Figures and Tables

**Table 1:** Hydrogen-bond geometry (Å, °)

*D*—H⋯*A*	*D*—H	H⋯*A*	*D*⋯*A*	*D*—H⋯*A*
N1—H1⋯O1^i^	0.86	2.50	3.340 (3)	166
C2—H2⋯O1^i^	0.93	2.43	3.251 (3)	147
C13—H13*A*⋯O1^i^	0.96	2.40	3.246 (3)	147

Symmetry code: (i) 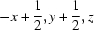.

**Fig. 1.**
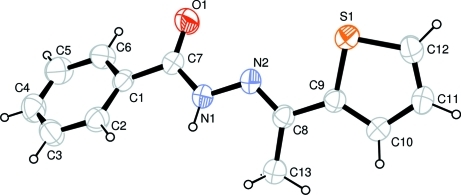
The molecular structure of the title compound with 40% probability displacement (arbitrary spheres for H atoms).

## supplementary crystallographic information

### Comment

The hydrazone derivatives has attracted our much attention because they have shown to be potential DNA damaging and mutagenic agents (Okabe *et al.*, 1993). As part of the ongoing investigation on the relationship between structure and property of hydrazone derivatives (Qiang *et al.*, 2007) the title compound has recently been prepared in our laboratory and its crystal structure is reported here.

The molecular structure of the title compound is shown in Fig. 1. In the molecule, the formohydrazide fragment is approximately co-planar [r.m.s deviation = 0.0146 Å] and the mean plane is oriented with respect to the phenyl ring and thiophene ring at 24.47 (11) and 28.86 (13)°, respectively. The N2—C8 bond length of 1.286 (2) Å shows a typical C═N double bond. The thiophene and benzamide units are located on the opposite sites of the C═N bond, showing an E configuration.

Intermolecular N—H···O and weak C—H···O hydrogen bonding is present in the crystal structure (Table 1).

### Experimental

Benzohydrazide (0.68 g, 5 mmol) was dissolved in ethanol (25 ml), then acetic acid (0.2 ml) was added to the ethanol solution with stirring. The solution was heated at about 333 K for several minutes until it became clear. 2-Acetylthiophene (0.63 g, 5 mmol) was then added slowly into the solution, and the mixture solution was refluxed for 6 h. After cooling to room temperature, yellow microcrystals appeared. The microcrystals were separated from the solution and washed with cold water three times. Recrystallization was performed twice with absolute methanol to obtain single crystals of the title compound.

### Refinement

H atoms were placed in calculated positions with C—H = 0.93 (aromatic), 0.96 Å (methyl) and N—H = 0.86 Å, and refined in riding mode with *U*_iso_(H) = 1.5*U*_eq_(C) for methyl H atoms and 1.2*U*_eq_(C,N) for the others.

### Crystal data

**Table:**

C_13_H_12_N_2_OS	*F*(000) = 1024
*M**_r_* = 244.31	*D*_x_ = 1.359 Mg m^−^^3^
Orthorhombic, *P**b**c**a*	Mo *K*α radiation, λ = 0.71073 Å
Hall symbol: -P 2ac 2ab	Cell parameters from 2153 reflections
*a* = 9.906 (3) Å	θ = 3.3–25.2°
*b* = 10.542 (5) Å	µ = 0.26 mm^−^^1^
*c* = 22.870 (5) Å	*T* = 294 K
*V* = 2388.3 (14) Å^3^	Block, yellow
*Z* = 8	0.32 × 0.29 × 0.28 mm

### Data collection

**Table:**

Rigaku R-AXIS RAPID IP diffractometer	1552 reflections with *I* > 2σ(*I*)
Radiation source: fine-focus sealed tube	*R*_int_ = 0.036
graphite	θ_max_ = 25.2°, θ_min_ = 3.3°
Detector resolution: 10.0 pixels mm^-1^	*h* = −10→11
ω scans	*k* = −11→12
7859 measured reflections	*l* = −27→23
2153 independent reflections	

### Refinement

**Table:**

Refinement on *F*^2^	Primary atom site location: structure-invariant direct methods
Least-squares matrix: full	Secondary atom site location: difference Fourier map
*R*[*F*^2^ > 2σ(*F*^2^)] = 0.041	Hydrogen site location: inferred from neighbouring sites
*wR*(*F*^2^) = 0.101	H-atom parameters constrained
*S* = 1.03	*w* = 1/[σ^2^(*F*_o_^2^) + (0.0465*P*)^2^ + 0.4798*P*] where *P* = (*F*_o_^2^ + 2*F*_c_^2^)/3
2153 reflections	(Δ/σ)_max_ = 0.001
155 parameters	Δρ_max_ = 0.20 e Å^−^^3^
0 restraints	Δρ_min_ = −0.17 e Å^−^^3^

### Special details

**Table:**

Geometry. All e.s.d.'s (except the e.s.d. in the dihedral angle between two l.s. planes) are estimated using the full covariance matrix. The cell e.s.d.'s are taken into account individually in the estimation of e.s.d.'s in distances, angles and torsion angles; correlations between e.s.d.'s in cell parameters are only used when they are defined by crystal symmetry. An approximate (isotropic) treatment of cell e.s.d.'s is used for estimating e.s.d.'s involving l.s. planes.
Refinement. Refinement of *F*^2^ against ALL reflections. The weighted *R*-factor *wR* and goodness of fit *S* are based on *F*^2^, conventional *R*-factors *R* are based on *F*, with *F* set to zero for negative *F*^2^. The threshold expression of *F*^2^ > σ(*F*^2^) is used only for calculating *R*-factors(gt) *etc*. and is not relevant to the choice of reflections for refinement. *R*-factors based on *F*^2^ are statistically about twice as large as those based on *F*, and *R*- factors based on ALL data will be even larger.

### Fractional atomic coordinates and isotropic or equivalent isotropic displacement parameters (Å^2^)

**Table:**

	*x*	*y*	*z*	*U*_iso_*/*U*_eq_	
S1	0.61206 (5)	0.31374 (5)	0.02223 (3)	0.0507 (2)	
N1	0.30582 (15)	0.50999 (16)	0.12073 (8)	0.0425 (5)	
H1	0.3095	0.5895	0.1290	0.051*	
N2	0.41317 (15)	0.45043 (16)	0.09296 (8)	0.0422 (4)	
O1	0.19122 (15)	0.32660 (14)	0.12656 (9)	0.0679 (5)	
C1	0.08229 (18)	0.51074 (18)	0.16290 (10)	0.0376 (5)	
C2	0.0570 (2)	0.6393 (2)	0.15591 (10)	0.0462 (6)	
H2	0.1126	0.6881	0.1321	0.055*	
C3	−0.0508 (2)	0.6949 (2)	0.18432 (11)	0.0549 (6)	
H3	−0.0675	0.7810	0.1794	0.066*	
C4	−0.1335 (2)	0.6245 (3)	0.21977 (11)	0.0583 (7)	
H4	−0.2050	0.6629	0.2393	0.070*	
C5	−0.1101 (2)	0.4972 (2)	0.22631 (12)	0.0606 (7)	
H5	−0.1664	0.4490	0.2501	0.073*	
C6	−0.0040 (2)	0.4405 (2)	0.19801 (10)	0.0495 (6)	
H6	0.0103	0.3538	0.2024	0.059*	
C7	0.19588 (19)	0.4411 (2)	0.13447 (10)	0.0423 (5)	
C8	0.5283 (2)	0.50629 (18)	0.09602 (10)	0.0377 (5)	
C9	0.63941 (18)	0.44513 (18)	0.06501 (9)	0.0377 (5)	
C10	0.7727 (2)	0.4768 (2)	0.06465 (11)	0.0488 (6)	
H10	0.8080	0.5451	0.0853	0.059*	
C11	0.8514 (2)	0.3953 (2)	0.02980 (11)	0.0549 (7)	
H11	0.9441	0.4043	0.0249	0.066*	
C12	0.7782 (2)	0.3029 (2)	0.00428 (11)	0.0509 (6)	
H12	0.8141	0.2409	−0.0201	0.061*	
C13	0.5569 (2)	0.6255 (2)	0.12900 (11)	0.0523 (6)	
H13A	0.5023	0.6930	0.1137	0.078*	
H13B	0.6506	0.6471	0.1249	0.078*	
H13C	0.5362	0.6130	0.1696	0.078*	

### Atomic displacement parameters (Å^2^)

**Table:**

	*U*^11^	*U*^22^	*U*^33^	*U*^12^	*U*^13^	*U*^23^
S1	0.0387 (3)	0.0543 (4)	0.0590 (4)	−0.0030 (3)	−0.0008 (3)	−0.0143 (3)
N1	0.0321 (9)	0.0425 (9)	0.0529 (13)	0.0001 (8)	0.0042 (8)	−0.0043 (8)
N2	0.0328 (9)	0.0455 (9)	0.0484 (12)	0.0030 (8)	0.0026 (8)	−0.0031 (8)
O1	0.0465 (9)	0.0458 (9)	0.1112 (16)	−0.0032 (7)	0.0204 (9)	−0.0110 (9)
C1	0.0319 (10)	0.0441 (11)	0.0368 (13)	−0.0032 (9)	−0.0021 (9)	−0.0031 (9)
C2	0.0422 (12)	0.0472 (12)	0.0491 (16)	−0.0032 (10)	0.0022 (11)	0.0017 (10)
C3	0.0555 (14)	0.0501 (13)	0.0591 (17)	0.0100 (11)	0.0014 (12)	−0.0064 (12)
C4	0.0485 (13)	0.0730 (17)	0.0533 (17)	0.0060 (13)	0.0099 (12)	−0.0136 (13)
C5	0.0592 (15)	0.0688 (16)	0.0538 (18)	−0.0099 (13)	0.0207 (13)	−0.0064 (12)
C6	0.0537 (13)	0.0479 (12)	0.0470 (15)	−0.0034 (11)	0.0064 (11)	−0.0016 (11)
C7	0.0344 (11)	0.0441 (12)	0.0485 (15)	−0.0015 (10)	−0.0023 (10)	−0.0020 (10)
C8	0.0345 (10)	0.0393 (10)	0.0392 (13)	0.0005 (9)	−0.0022 (10)	0.0023 (9)
C9	0.0346 (10)	0.0382 (10)	0.0402 (13)	0.0003 (9)	−0.0007 (9)	0.0029 (9)
C10	0.0375 (11)	0.0448 (12)	0.0641 (17)	−0.0057 (10)	0.0032 (11)	−0.0064 (11)
C11	0.0354 (11)	0.0549 (13)	0.0744 (19)	0.0005 (10)	0.0097 (12)	−0.0043 (13)
C12	0.0437 (12)	0.0548 (14)	0.0542 (16)	0.0068 (11)	0.0061 (11)	−0.0068 (12)
C13	0.0389 (11)	0.0533 (13)	0.0646 (18)	−0.0006 (10)	0.0028 (11)	−0.0126 (12)

### Geometric parameters (Å, °)

**Table:**

S1—C12	1.700 (2)	C4—H4	0.9300
S1—C9	1.717 (2)	C5—C6	1.371 (3)
N1—C7	1.346 (2)	C5—H5	0.9300
N1—N2	1.389 (2)	C6—H6	0.9300
N1—H1	0.8600	C8—C9	1.459 (3)
N2—C8	1.286 (2)	C8—C13	1.493 (3)
O1—C7	1.222 (2)	C9—C10	1.361 (3)
C1—C6	1.387 (3)	C10—C11	1.407 (3)
C1—C2	1.388 (3)	C10—H10	0.9300
C1—C7	1.492 (3)	C11—C12	1.347 (3)
C2—C3	1.380 (3)	C11—H11	0.9300
C2—H2	0.9300	C12—H12	0.9300
C3—C4	1.371 (3)	C13—H13A	0.9600
C3—H3	0.9300	C13—H13B	0.9600
C4—C5	1.370 (3)	C13—H13C	0.9600
			
C12—S1—C9	92.23 (10)	O1—C7—C1	121.45 (18)
C7—N1—N2	118.85 (17)	N1—C7—C1	116.53 (18)
C7—N1—H1	120.6	N2—C8—C9	116.11 (18)
N2—N1—H1	120.6	N2—C8—C13	125.55 (19)
C8—N2—N1	116.57 (17)	C9—C8—C13	118.34 (17)
C6—C1—C2	118.51 (19)	C10—C9—C8	128.73 (19)
C6—C1—C7	117.01 (18)	C10—C9—S1	110.31 (16)
C2—C1—C7	124.48 (19)	C8—C9—S1	120.95 (14)
C3—C2—C1	120.0 (2)	C9—C10—C11	113.0 (2)
C3—C2—H2	120.0	C9—C10—H10	123.5
C1—C2—H2	120.0	C11—C10—H10	123.5
C4—C3—C2	120.7 (2)	C12—C11—C10	112.8 (2)
C4—C3—H3	119.6	C12—C11—H11	123.6
C2—C3—H3	119.6	C10—C11—H11	123.6
C5—C4—C3	119.6 (2)	C11—C12—S1	111.59 (17)
C5—C4—H4	120.2	C11—C12—H12	124.2
C3—C4—H4	120.2	S1—C12—H12	124.2
C4—C5—C6	120.3 (2)	C8—C13—H13A	109.5
C4—C5—H5	119.8	C8—C13—H13B	109.5
C6—C5—H5	119.8	H13A—C13—H13B	109.5
C5—C6—C1	120.8 (2)	C8—C13—H13C	109.5
C5—C6—H6	119.6	H13A—C13—H13C	109.5
C1—C6—H6	119.6	H13B—C13—H13C	109.5
O1—C7—N1	121.93 (19)		

### Hydrogen-bond geometry (Å, °)

**Table:**

*D*—H···*A*	*D*—H	H···*A*	*D*···*A*	*D*—H···*A*
N1—H1···O1^i^	0.86	2.50	3.340 (3)	166
C2—H2···O1^i^	0.93	2.43	3.251 (3)	147
C13—H13A···O1^i^	0.96	2.40	3.246 (3)	147

Symmetry codes: (i) −*x*+1/2, *y*+1/2, *z*.
